# Psychosis and the Dissonance in the Doctor-Patient Relationship; a Thematic Analysis

**DOI:** 10.1192/bjo.2022.208

**Published:** 2022-06-20

**Authors:** Aimee Lawton

**Affiliations:** The University of Edinburgh, Edinburgh, United Kingdom

## Abstract

**Aims:**

Within psychiatry, relationships between doctors and patients with psychosis are significant determinants of attitudes, adherence, and therapeutic outcomes. Current research focuses on communication within psychiatrist-patient interactions with limited evaluation of the patient's perspective. Understanding the components underpinning the patient's relationship with their doctor could help improve outcomes for individuals with psychosis.

**Methods:**

Eight participants, recruited through advocacy programmes, were interviewed. All had a diagnosis of psychosis or its subtypes. Interviews lasted between forty and eighty minutes. Thematic analysis of semi-structured interviews allowed exploration of important themes within doctor-patient relationships. Ethical procedures were implemented in accordance with British Psychological Society guidelines.

**Results:**

Participants’ narratives identified three salient themes perceived to influence doctor-patient relationships. Participants explored ‘Interactions with Medical Professionals’, focusing on communication and discussion styles. Doctors were not perceived as empathic, open listeners, reducing trust and limiting conversation during interactions. Participants described reduced engagement due to perceived misunderstanding and highlighted the impact of time constraints, guidelines, and limited medical training on relationships.

Secondly, participants discussed the ‘Diagnostic Process’, suggesting it had a negative influence on the relationship due to delivery methods.

Finally, participants explored ‘Treatment’, highlighting an overwhelming reliance on medication, lack of explanations, and lack of psychological therapies, which contradicted with patients’ preferences.

**Conclusion:**

The narratives describe a relationship in which patients feel misunderstood, furthering patient disengagement and resulting in a vicious cycle of dissonance that limits health outcomes. Findings suggest a need to incorporate psychological therapies into doctor-patient interactions to allow increased communication and understanding.

